# The translational potential of human induced pluripotent stem cells for clinical neurology: The translational potential of hiPSCs in neurology

**DOI:** 10.1007/s10565-016-9372-7

**Published:** 2016-12-03

**Authors:** Helen Devine, Rickie Patani

**Affiliations:** 1 Department of Molecular Neuroscience, UCL Institute of Neurology, Queen Square, London, WC1N3BG UK; 2 Sobell Department of Motor Neuroscience and Movement Disorders, UCL Institute of Neurology, Queen Square, London, UK; 3 National Hospital for Neurology and Neurosurgery, UCL Institute of Neurology, Queen Square, London, WC1N 3BG UK; 4 Department of Clinical Neurosciences, University of Cambridge, Cambridge, UK; 5 Euan MacDonald Centre for MND, University of Edinburgh, Edinburgh, UK

**Keywords:** Cellular therapy, Disease modeling, Drug discovery, Human induced pluripotent stem cells (hiPSCs), Neurology, Translational medicine

## Abstract

The induced pluripotent state represents a decade-old Nobel prize-winning discovery. Human-induced pluripotent stem cells (hiPSCs) are generated by the nuclear reprogramming of any somatic cell using a variety of established but evolving methods. This approach offers medical science unparalleled experimental opportunity to model an individual patient’s disease “in a dish.” HiPSCs permit developmentally rationalized directed differentiation into any cell type, which express donor cell mutation(s) at pathophysiological levels and thus hold considerable potential for disease modeling, drug discovery, and potentially cell-based therapies. This review will focus on the translational potential of hiPSCs in clinical neurology and the importance of integrating this approach with complementary model systems to increase the translational yield of preclinical testing for the benefit of patients. This strategy is particularly important given the expected increase in prevalence of neurodegenerative disease, which poses a major burden to global health over the coming decades.

## Introduction

Medicine has evolved a sophisticated taxonomic repertoire that is based upon particular constellations of predominantly clinical and macroscopic/imaging features. Although such an approach has clear utility in medical practice, it leaves unresolved to some extent the cellular and molecular basis of disease. It follows that a complementary taxonomic (re)classification to reflect underlying—and potentially therapeutically targetable—molecular mechanisms may serve to strengthen both our diagnostic and therapeutic capacities. In order to first gain accurate insight into the molecular basis of human disease, it is crucial to employ an integrated approach that recognizes inherent limitations in each model system when used in isolation. Animal models and human non-neuronal cell lines have provided invaluable insight into developmental and translational neuroscience. Yet their success in identifying novel and clinically impactful therapies has been underwhelming, perhaps reflecting an inability to capture the true complexity of human neurological disease by using these approaches in isolation. Advances over the last decade have transformed the landscape of mechanistic evaluation and drug discovery in neurodegenerative disease, with new technologies permitting the study of previously inaccessible human cellular subtypes. The induced pluripotent state represents a Nobel prize-winning discovery made in the laboratory of Shinya Yamanaka in Kyoto, Japan, in 2006 and 2007 (Takahashi and Yamanaka 2006; Takahashi et al. 2007). Human-induced pluripotent stem cells (hiPSCs) are generated by the nuclear reprogramming of any somatic cell using either genome integrating or non-integrating “footprint-free” methods (reviewed in Gonzalez et al. 2011). This approach offers medical science considerable and unprecedented experimental opportunities to model an individual patient’s disease “in a dish.” HiPSCs permit ontogeny-recapitulating directed differentiation into any human cell types, which themselves express donor cell mutation(s) at pathophysiological levels and thus hold considerable potential for disease modeling, drug discovery and potentially cell-based therapies.

## Modeling neurodegeneration: the role of hiPSCs

**Fig. 1:**
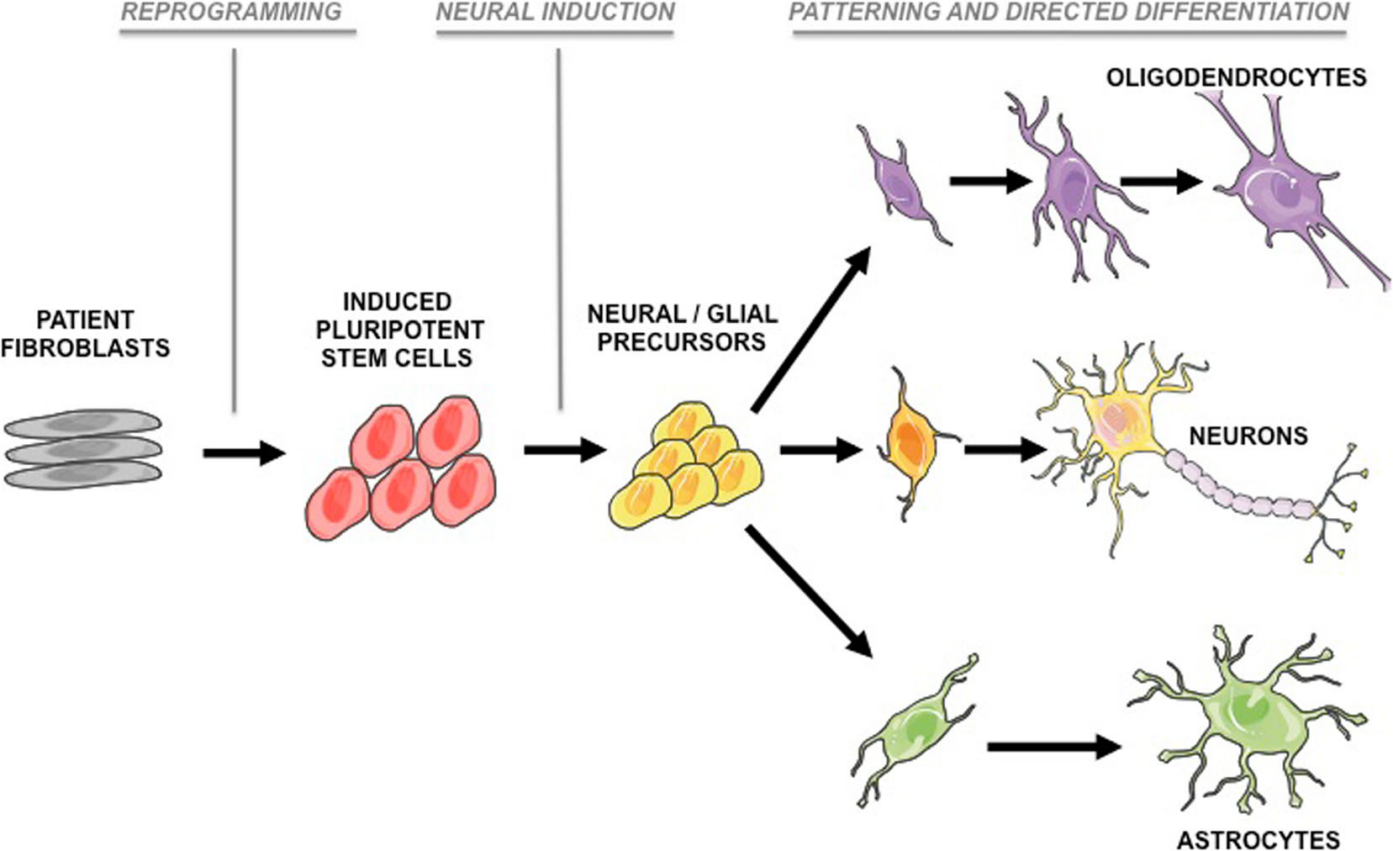
A schema depicting the generation of human-induced pluripotent stem cells from patient fibroblasts followed by sequential phases of lineage restriction. Directed differentiation paradigms can generate region-specific neural precursors, which can subsequently be differentiated into neurons, astrocytes, and oligodendrocytes. Diagrams were drawn using templates freely available from Servier Medical Art (http://www.servier.co.uk/content/servier-medical-art)

The failure in clinical translation from preclinical models is particularly evident in the field of neurodegenerative disease, possibly arguing for the requirement of a human experimental system to complement—*but not replace*—existing models. While human postmortem tissue provides a valuable resource for investigating the end-stage pathological processes in a clinical disease, it does not allow dynamic insight into initiating molecular pathogenic events. HiPSCs possess two defining attributes: (i) the ability to self renew and (ii) the capacity to differentiate into any of the cell types comprising the organism from which they are derived. However, with this potential comes the complexity of *directing* differentiation into highly refined and regionally specified subtypes of neurons and glia (see Fig. 1).

This paradigm utilizes insights from developmental biology to rationalize a program of stage-specific morphogenetic instructions in order to predictably manipulate cell fate to desired lineages. These attributes—when considered with the fact that one can generate patient-specific hiPSCs—justify current enthusiasm about this technology to help elucidate cellular and molecular determinants of human disease. One major utility of this model is to resolve the functional cellular and molecular sequelae of monogenetic diseases, an approach that has now been validated by myriad studies including adult-onset conditions. Indeed, well-characterized and developmentally rationalized protocols for neuronal and glial specification (reviewed elsewhere; Zirra et al. 2016; Goldman and Kuypers 2015; Tyzack et al. 2016; Patani 2016) have been employed on a range of patient-derived hiPSCs for both monogenic and sporadic neurodegenerative diseases to define disease-related phenotypes (see Table 1 for representative examples). The differentiation of clearly defined and functional cellular subtypes from stem cells provides the opportunity not only to elucidate but also to put into precise sequence the molecular steps culminating in cellular demise by conducting time-resolved experiments. However, in patients, these cells do not exist in isolation and, therefore, developing incrementally complex neuronal circuits, neuron-glia co-culture paradigms, and complex 3D “organoids” are likely to deepen our understanding of these diseases (reviewed in Clevers 2016). RNA sequencing has been used to identify potential pathological pathways in an unbiased manner and, coupled with gene editing to correct a mutation, provide evidence that phenotypes detected are mutation-dependent (Kiskinis et al. 2014; Reinhardt et al. 2013). Indeed, an experimental workflow for such an approach has recently been proposed (Merkle and Eggan 2013). Identification of early pathological processes—including post-transcriptional mechanisms of disease (Patani et al. 2012b)—offers the prospect therapeutic target definition for intervention at a presymptomatic/early symptomatic stage.

**Table 1:** Phenotypes captured of major neurological diseases

Disease	Genes	Cell type	Phenotype(s) detected/treatment if attempted	Ref.
Movement disorders				
Parkinson’s disease	*PARKIN*	Midbrain dopaminergic (mDA) neurons	Shorter neurite length and microtubule instability. Reversed with overexpression of PARKIN or treatment with microtubule-stabilizing drug taxol.	(Ren et al. 2015)
	*LRRK2*	Neural progenitors and neurons (mDA and non-mDA).	Increased levels of mitochondrial DNA damage, corrected with gene editing via zinc finger nuclease technology. Phenotype only detected in neurons (including from presymptomatic patients), but not in fibroblasts or iPSCs.	(Sanders et al. 2014)
	*SCNA* triplication	mDA neurons	Increased levels of α-synuclein and susceptibility to cellular oxidative stress.	(Devine et al. 2011)
Gaucher’s disease	*GBA1* (strongest genetic risk factor for PD)	mDA neurons	Increased levels of α-synuclein, reduced dopamine storage and reuptake in patients with parkinsonism. Reversed with glucocerebrosidase chaperone.	(Aflaki et al. 2016)
	*GBA1*	mDA neurons	Abnormal calcium handling and increased vulnerability to stress. Autophagy defects.	(Schondorf et al. 2014)
Huntington’s disease	CAG repeat (72) in *HTT* gene	Striatal neurons	Increased caspase activity with growth factor deprivation in neural stem cells.	(Zhang et al. 2010)
Neuro-muscular disease				
Duchenne muscular dystrophy	*DYSTROPHIN*	Myoblasts	Undetectable levels of dystrophin protein in mutant myoblasts. Decreased myotube formation compared to controls. Aberrant intracellular signaling.	(Choi et al. 2016)
Motor neuron disease	*TARDBP*	Spinal motor (spM) neurons	TDP-43 proteinopathy, cell-specific vulnerability.	(Bilican et al. 2012)
	*TARDBP*	Astrocytes	Cytoplasmic mislocation of TDP-43. Reduced survival under basal conditions.	(Serio et al. 2013)
Spinal muscular atrophy	*SMN1*	spM neurons	Lack of SMN1 expression in spM neurons from patients with SMA. Disease phenotype of spM neuron loss.	(Ebert et al. 2009)
	*SMN1*	Purified spM neurons	Elevated ER stress.	(Ng et al. 2015)
Spino-bulbar muscular atrophy	CAG repeat in androgen receptor gene	spM neurons	Increased acetyl α-tubulin.	(Grunseich et al. 2014)
Dementia				
Alzheimer’s disease	*PRESENILIN 1 AND 2*	Cortical neurons	Increased levels of amyloid β.	(Yagi et al. 2011)
	Familial: (APP)	Cortical neurons, astrocytes	Stress phenotypes with intracellular amyloid β. Heterogeneity and phenotypic differences between familial and sporadic AD.	(Kondo et al. 2013)
Fronto-temporal dementia	*C9ORF72*	Cortical neurons	Increased sensitivity to cellular stress with autophagy inhibitors.	(Almeida et al. 2013)
Other neurological disorders				
Monge’s disease		Cortical neurons	Decreased excitability in neurons of patients with chronic mountain sickness (migraine, confusion, fatigue, memory loss). Decreased sodium channel expression.	(Zhao et al. 2015)
Dravet syndrome	*SCN1A*	Forebrain neurons (bipolar and pyramidal)	Increased sodium currents using whole cell voltage and current clamp recordings. Reduced threshold for action potential firing.	(Liu et al. 2013)
Rett syndrome	*RTT*	Neurons	Altered morphology, reduced glutamatergic synapse number. Altered calcium transients suggesting a deficiency in neuronal network connectivity.	(Marchetto et al. 2010)

## Maturational status of terminally differentiated neurons and glia

We and others have studied the maturity of terminally differentiated pluripotent stem cell-derived region-specific neurons using genome-wide gene expression and splicing analysis and found that they represent a fetal developmental state when compared to isotopic but anisochronic somatic counterparts (Patani et al. 2012a; Miller et al. 2013; Ho et al. 2016). This finding may bring into question their ability to model adult onset conditions. Yet, patient-specific hiPSCs have now been robustly shown across a range of neurogenetic conditions—summarized in Table 1—to possess the capacity to capture early pathogenic events in a mutation- and cell type-dependent manner. The ability to model adult-onset conditions in what is essentially a developmental system can be reconciled by the possibility that the in vivo environment (glial-neuronal interaction for example) may help to compensate cell autonomous neuronal dysfunction. Recognizing the ability of hiPSC derivatives to capture disease-relevant phenotypes then raises the interesting issue of how early the presymptomatic phase may actually begin (i.e., the duration of “compensated neuronal dysfunction”). Indeed, important insights have been gained by studying even neural precursors in certain familial forms of schizophrenia (Yoon et al. 2014), familial dysautonomia (Lee et al. 2012; Lee et al. 2009), and hereditary spastic paraparesis (Mishra et al. 2016), reinforcing the utility of this approach for elucidation of developmental phenotypes in pre-manifest disease states. In other cases, differentiated cell types have been exposed to stressors in order to elicit a phenotype (Reinhardt et al. 2013; Nguyen et al. 2011; Donnelly et al. 2013).

Can we do anything to accelerate aging of hiPSC-derived neurons so they more faithfully represent their adult counterparts? Epigenomic changes including those in transcriptional and chromatin networks are observed with aging (Booth and Brunet 2016). Therefore, to comprehensively study neurodegenerative disease, it may be a complementary strategy to induce aging in hiPSC models as a useful comparator. To this end, experiments from Lorenz Studer’s group have demonstrated that progerin-induced aging of hiPSC-derived midbrain dopaminergic neurons to a more adult-like state is achievable (Miller et al. 2013). A more recent study from Fred Gage’s laboratory systematically compared hiPSC-derived neurons with direct conversion/‘transdifferentiation’ from patients across a diverse age range to confirm that reprogramming “resets” age, while transdifferentiation preserves donor age, thus highlighting the complementarity of these paradigms to study age-dependent phenotypes (Mertens et al. 2015). Indeed, direct conversion to multiple region-specific and clinically relevant neuronal and glial subtypes has also been achieved (Son et al. 2011; Caiazzo et al. 2011; Yang et al. 2013a; Caiazzo et al. 2015), reinforcing the practical feasibility of comparative studies between direct conversion and hiPSC directed differentiation paradigms. A further interesting approach to inducing/accelerating aging of hiPSC-derived sympathetic neurons was their functional connection with cardiomyocytes (Oh et al. 2016). This study harnessed developmentally rationalized directed differentiation and in vivo circuitry to foster maturation status.

## Genomic variation between hiPSC lines

Line-to-line variability continues to be a concern among stem cell biologists. However, significant advances in gene editing through zinc finger nucleases (ZFNs, Wood et al. 2011), transcriptional activator-like effector nucleases (TALENs, Boch et al. 2009), and more recently the development of the clustered regularly interspaced short palindromic repeats (CRISPR)-associated system (CRISPR/Cas9, Cong et al. 2013) allow the generation of isogenic controls to increase confidence in identifying mutation-dependent cellular and molecular phenotypes. These approaches and their practical utility in patient-specific hiPSCs have been recently reviewed elsewhere (Hendriks et al. 2016). Genome editing technologies are particularly relevant to monogenic diseases rather than complex genetic disorders or sporadic disease (Shribman et al. 2013; Samani et al. 2015; Patani et al. 2013; Athappily et al. 2013). An ideal approach for mendelian disorders is to generate reciprocal isogenic lines for a given mutation being studied—i.e., the mutation is corrected to create one isogenic pair, and it is separately inserted into a control line to generate a second isogenic pair (Liu et al. 2012). An alternative approach to genome editing is to utilize larger numbers of control and mutant lines, but this has the potential to become prohibitively time- and resource-consuming, although feasibility has already been demonstrated in the context of a large and cohesive consortium (Consortium 2012).

Generating isogenic controls, standardizing optimal differentiation protocols, and reproducing mutation-dependent phenotypes *across different laboratories* are important considerations in future work. Additionally, intensive characterization of several lines derived from ethnically diverse control cases also has value to serve as a reference point, particularly if these lines are made available to other investigators. Such an approach would not only provide an invaluable resource but would also significantly reduce costs for stem cell researchers. Indeed, the hiPSC field has begun to achieve such stem cell repositories and had the foresight to include “secondary products” (e.g., reporter lines). This strategy promotes data sharing and comparison. Further attributes including integration-free lines, equal male and female line representation, and the prospect of intensively characterized cryopreservable differentiated precursors make such stem cell repositories an attractive prospect. Such non-profit initiatives also stand to benefit from close collaboration with industry.

## Compound screening and drug discovery

Drug development is expensive and time-consuming, and those therapeutics that do emerge from preclinical studies have a low conversion rate into successful disease treatments due to limitations with either safety or efficacy. This failure of translation is particularly evident in the field of neurodegeneration, where only a very small percentage of drugs that reach the development phase are actually ever marketed (Ringel et al. 2013; Rubin 2008). Some putative reasons for this failure include (i) selection of diseases where the molecular pathogenesis is poorly understood, this precluding a mechanistically rationalized approach (ii) over reliance on one model system or the use of models that have poor predictive value of clinical success (e.g., due to interspecies differences, Peng et al. 2013; Seok et al. 2013; Scannell et al. 2012), (iii) candidate compounds that have poor safety profiles or (iv) pharmacodynamic properties (e.g., do not penetrate the blood-brain barrier), and (v) clinical trial design that is suboptimal due to patient heterogeneity and/or lack of appropriate end point measures.

**Fig. 2:**
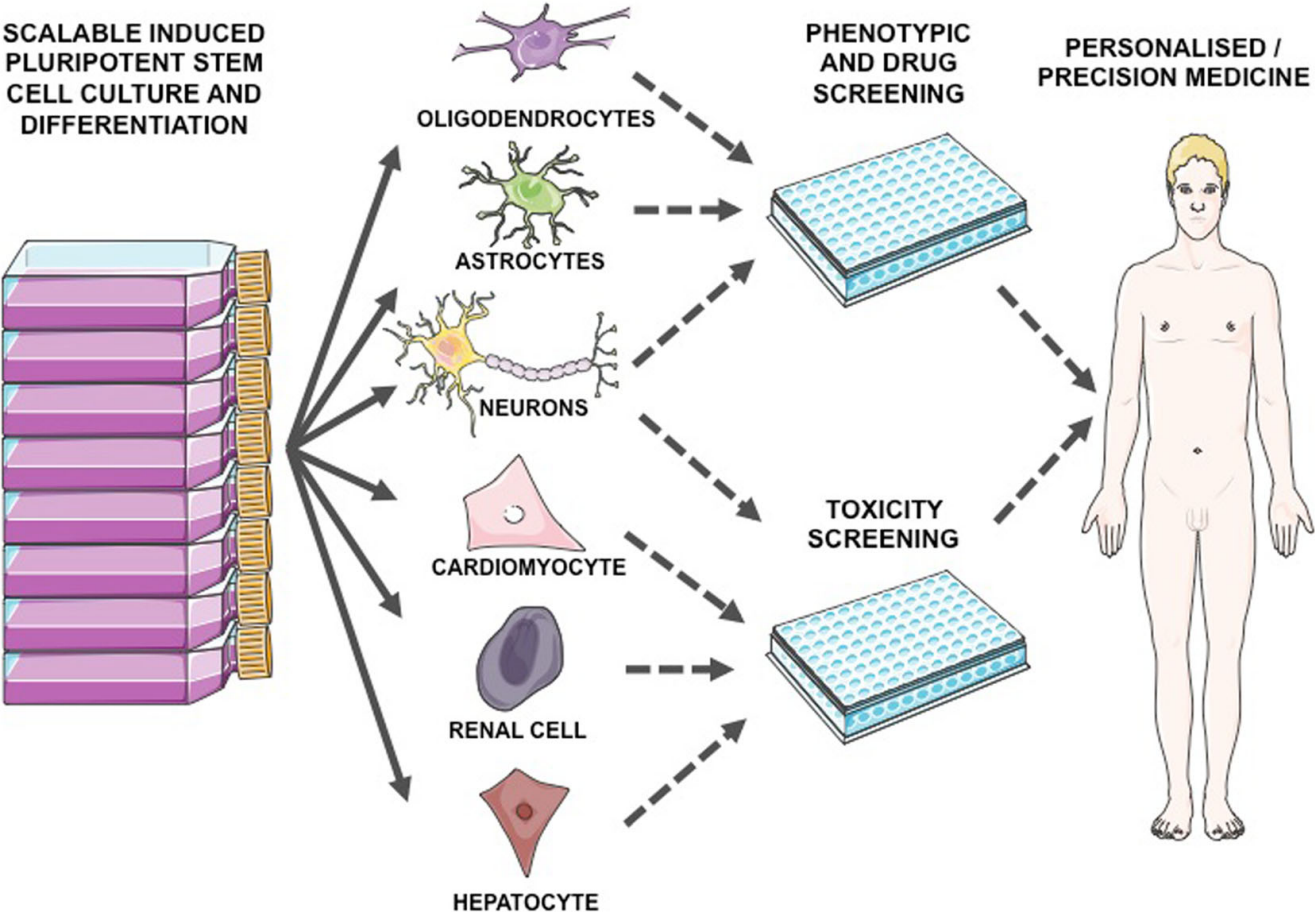
A *workflow* for using human-induced pluripotent stem cell derivatives for disease modeling, drug discovery, and toxicity assays in high throughput. Diagrams were drawn using templates freely available from Servier Medical Art (http://www.servier.co.uk/content/servier-medical-art)

The historical inaccessibility to most adult human neural cell types has consequently prevented their experimental integration into preclinical testing. In the last decade since their discovery, hiPSC models have already successfully identified promising compounds preclinically, some of which have since entered clinical trials (Wainger et al. 2014; Kiskinis et al. 2014). It is also noteworthy that drugs, which previously failed in expensive clinical trials, have also been shown to fail in relevant hiPSC models (Yang et al. 2013b), highlighting their utility/predictive power in preclinical testing. High throughput assays of patient-derived neurons and glia therefore offer the potential to transform preclinical testing when integrated with current in vivo approaches (see Fig. 2). Furthermore, due to one of their defining attributes of potentially limitless self-renewal, there is a theoretically unlimited supply of cellular material. Indeed, 3D culture techniques have been shown to allow the maturation of billions of uniform cells in a single flask (Rigamonti et al. 2016). Such scalability will in turn allow more comprehensive optimization of dosing regimen as well as concentration and duration of treatment through systematic fine-tuning, rather than the somewhat arbitrary dosing regimen currently in place for many drugs used in clinical medicine.

Characterizing a population of cryopreservable intermediate neural derivatives (e.g., region-specific precursors) would further improve scalability of cultures for high-content cell-based screening approaches. High-throughput screening has been used to identify compounds that can optimize directed differentiation of hiPSCs (Han et al. 2009; Maury et al. 2015) and demonstrates practical feasibility of employing such approaches for drug discovery. Indeed, this method has revealed important cell type- and species-specific effects, both in terms of toxicity and neuroprotective effects (Peng et al. 2013). A recent study performed a large-scale compound screen with hiPSC-derived neural precursor cells to decrease the Zika virus infection, demonstrating the utility of high-throughput approaches at the neural precursor stage (Xu et al. 2016). Some further representative examples of studies that have exploited hiPSC-derived neural precursors and/or neurons for drug discovery and toxicity assessment are provided in Table 2 below. The repertoire of live content readouts is steadily increasing; robust and commercially available assays include live/dead, neurite length/complexity, and mitochondrial integrity, to mention a few. The prospect of multiplexing these parameters in a human neuronal system is attractive from a disease modeling perspective.

**Table 2:** Drug screening of patient-specific iPSC derivatives

Disease	Drug	Cell type	Outcome	Ref.
Familial dysautonomia	6912 compounds tested; 8 hits	Neural crest lineage precursors	Alpha-2 adrenergic receptor activity implicated in regulating IKBKAP expression. SKF-86466 induced IKBKAP transcription through regulation of intracellular cAMP levels/PKA-dependent CREB phosphorylation. Restored expression of autonomic neuron markers.	(Lee et al. 2012)
Gaucher’s/Parkinson’s disease	NCGC607, non-inhibitory chaperone of glucocerebrosidase	mDA neurons	Restored glucocerebrosidase activity and reduced α-synuclein levels.	(Aflaki et al. 2016)
Motor neuron disease (TARDBP)	Trichostatin A (a histone deacetyltransferase inhibitor) Spliceostatin A (a spliceosomal factor inhibitor). Anacardic acid and garcinol (histone acetyltransferase inhibitors)	spM neurons	Gene expression analysis suggested transcription and RNA splicing altered in ALS MN. Anacardic acid reduced arsenite-induced death compared with non-treated, reduced TDP-43 mRNA expression and increased length of neurites.	(Egawa et al. 2012)
Motor neuron disease (SOD1)	5000 compounds at 2 concentrations. Kenpaullone identified. Also trialed dexpramipexole—failed phase III clinical trials.	spM neurons	Increased number of surviving spM neurons. 9 compounds identified and particularly kenpaullone, a dual-kinase inhibitor. Kenpaullone promoted survival and supported the morphology and function of the spM neurons in SOD1 mouse model. Subsequently shown to promote survival in MND iPSC-derived spM neurons. Dexpramipexole: no improvement in survival.	(Yang et al. 2013b)
Spinal muscular atrophy	Valporic acid and tobramycin	spM neurons	Treated spM neurons demonstrated 2–3× increased SMA protein compared with untreated.	(Ebert et al. 2009)
	Small-molecule inhibitors of ER stress: 4-phenylbutyrate, kifunensine, salubrinal, guanabenz, and GSK2606414.	spM neurons	Success assessed by spM neuron survival and stress response. Cell culture model accurately predicted in vivo response in SMA mice with guanabenz most successfully.	(Ng et al. 2015)
Alzheimer’s disease	Compound E (γ-secretase inhibitor) Compound W (selective Aβ42 reduction)	Cortical neurons	Dose-dependent reduction in Aβ42 and Aβ40 with compound E. Decrease in ratio of Aβ42 to Aβ40 with compound W.	(Yagi et al. 2011)
		Cortical neurons	Scalable high-throughput model for targeting tau aggregation model.	(Medda et al. 2016)
Fragile X syndrome	4000 Compounds tested	Neural stem cells, validation in neurons	FMRP product of FMR1 gene assay developed. Levels inversely proportional to clinical severity of patient. Identified 6 compounds able to, at least partially, reactivate FMR1 gene in primary screen and then validated in NSCs and neurons at different concentrations. Tibrofan: positive response in neurons.	(Kumari et al. 2015)
	50,000 compounds tested to reactivate Fmr1 gene.	Neuronal precursors	Found several compounds induced weak expression of fragile X mental retardation protein.	(Kaufmann et al. 2015)
Toxicity studies using hiPSC-derived neurons	Tested library of 80 compounds on 384-well plates with a 6-point concentration range.	Neurons β-III tubulin/MAP2 positive	Specifically looked at toxic effect on neurite outgrowth. Identified 6 compounds known to be neurotoxic.	(Ryan et al. 2016)
	2000 compounds	Neuronal precursors	Compared findings with rat cortical neurons to identify selective toxicity. Confirmed findings in second screen using hiPSC-derived neurons and fetal astrocytes with >80% showing cell specific toxicity.	(Malik et al. 2014)

Some drug discovery efforts have focused on “repurposing” FDA-approved drugs. This approach allows significant reduction of time to clinical use. Conversely, a 5–8-year period is required from identifying a hit using conventional compound library screening due to further necessary medicinal chemistry/drug optimization. It is likely that in many diseases, particularly degenerative processes, combinations of drugs may prove necessary for optimal therapeutic effect. From this perspective, the field of neurodegeneration can glean some relevant insights into therapeutic strategy from cancer biology. Furthermore, different drugs might be required either sequentially and/or combinatorially at distinct pathological “phases” within the context of a disease (e.g., relapsing-remitting vs. secondary progressive multiple sclerosis). Within defined phenotypes at a particular disease phase, it is important to experimentally resolve the crucial pathogenic event(s) in order to guide therapy development around such validated targets specifically.

The approach of screening compound libraries is important to inform which mechanistic phenomena are therapeutically manipulable. Important issues to address in the future (over and beyond the aforementioned stage-specific and combinatorial therapies) include screening compounds on enriched populations of specific cell types in mono- and co-culture. Furthermore, systematic approaches to optimizing the duration of therapy, drug concentration, and timing of initiation relative to disease phase are all important considerations. Integrated medicinal chemistry expertise is then crucial to optimize the compound for clinical trial use. To this end, it is essential to ensure that any potential compound is safe in humans, which highlights another role for hiPSC derivatives as a predictive cell-based model for toxicity assessment. HiPSCs themselves do not faithfully capture the physiological attributes of clinically relevant somatic cell types (e.g., those of the liver, kidney, heart, and brain) and thus cannot serve as appropriate model for predictive toxicity assessment. However, using ontogeny-recapitulating directed differentiation of hiPSCs to desired cell types allows more accurate toxicity assessment in a relevant cellular context (see Fig. 2). Drug development attrition rates are high as current assays do not always reflect damage to hepatic, renal, cardiac, and neuronal cells at least partially due to species differences in metabolism. Hepatocytes from donor tissue have been used to model drug toxicity; however, they are limited by scarcity of donor tissue, and high-quality tissue which is available is used—quite rightly—for donation (Greenhough et al. 2010). Hepatocyte-like cells have been differentiated from hiPSCs with success by several groups (Siller et al. 2015). A generic hepatocyte library to test therapeutic drugs in high-throughput screening for liver toxicity would be an invaluable resource, along with similar approaches for other aforementioned organ systems. Optimized rapid differentiation protocols in the future may offer the opportunity for personalized toxicity screening, which in turn would permit the formulation of a bespoke therapeutic strategy accounting for a patient’s own genetic polymorphisms.

## Cellular therapy

Strategies for generating patient-specific neuronal and glial subtypes—through either reprogramming and directed differentiation or direct conversion methods—have fuelled excitement about the prospect of cellular therapy to restore structure and function in neurodegenerative diseases. Issues of safety are paramount in this context and broadly include rigorous tumorogenicity and immunogenicity testing, recently reviewed elsewhere (Xie and Tang 2016; Neofytou et al. 2015). Additionally practical feasibility includes assuring authenticity of cell fate, scalability, and enrichment (e.g., generating a billion cells >95% enriched for a particular neuronal subtype would be an approximate manufacturing benchmark). There are broadly two cellular sources that one can consider—autologous (theoretically removing the need for immunosuppression) or allogeneic/“HLA matched.” It is also important to consider the goal of therapy at the cellular/molecular level—restoration of structure and function will require ontogeny-recapitulating differentiation of the cellular graft (e.g., authentic midbrain dopaminergic neurons in the case of Parkinson’s disease). Conversely, implanted cells may invoke/strengthen endogenous mechanisms of repair through local effects on neighboring cells. Geron Corporation initiated the first human stem cell trial for spinal cord injuries in 2010, but unfortunately this was discontinued shortly afterwards for business-related strategic (rather than scientific) reasons (Scott and Magnus 2014; Lebkowski 2011). In order to illustrate the relevant principles of cellular therapy here, we have considered two examples in some detail; Parkinson’s disease and age-related macular degeneration. Additional examples of relevant studies utilizing human iPSC-based cellular therapies for neurological disorders are then summarized in Table 3.

**Table 3:** Cellular therapies using iPSC derivatives

Disease	Host	Implanted cell type	Mode of implantation	Findings	Ref.
Multiple sclerosis	Mouse with experimental autoimmune encephalomyelitis	hiPSC neural stem cell	Intraventricular injection (lateral ventricle)	Donor cells localized to lesions. Increased remyelination and motor function.	(Zhang et al. 2016)
Progressive multiple sclerosis	Mouse and marmoset	hiPSC-derived oligodendrocytes	Intracerebral injection	Donor cells migrate to lesions and remyelinate axons.	(Thiruvalluvan et al. 2016)
Parkinson’s disease	Cynomologous monkey (*n* = 3, MPTP treated)	Autologous iPSC-derived dopaminergic midbrain neurons	Transplantation into putamen	Case 1: increased motor activity and graft survival. Cases 2 and 3: no improvement, poor graft survival.	(Hallett et al. 2015)
Stroke	Sprague Dawley and nude rats post-30 min distal MCA occlusion	hiPSC-derived cortical neuron-fated cells compared with non-fated.	Stereotactic intracerebral transplantation 48 h after MCA occlusion	Migration of cells to lesion. Function improved with cortical neuron-fated donor cells.	(Hallett et al. 2015)
Huntington’s disease	Rat with striatal degeneration induced by quinolinic acid	Mouse-derived iPSCs	Intraventricular injection (left lateral ventricle)	Improved learning and memory, increased metabolic activity and size of striatum. Graft differentiated into both neurons and astrocytes.	(Mu et al. 2014)
Motor neuron disease	SOD1G39A mice	hiPSC-derived neural stem cells with high aldehyde dehydrogenase activity and expression of integrin VLA4; Positive for LewisX-CXCR4-β1-integrin.	Repeated intrathecal or IV injection	Donor cells migrate and engraft. Improved neuromuscular function, increased spM neurons, and extended survival. Graft inhibited astrocyte activation.	(Nizzardo et al. 2014) (Nizzardo et al. 2016)
	12 patients with ALS initial cohort 6 patients with adapted injection device and lumbar stabilization	Fetal neural stem cells	Intraspinal injection	Phase 1 clinical trial. Well-tolerated, targeted to cervical and lumbar spinal cord segments.	(Glass et al. 2012) (Feldman et al. 2014)

There is a significant history to cellular therapy in Parkinson’s disease, particularly in the 1-methyl-4-phenyl-1,2,3,6-tetrahydropyridine (MPTP) primate model, which exhibits a characteristic loss of midbrain dopaminergic neurons in the substantia nigra and phenocopies the human condition. Transplantation of human fetal neural stem cells into this model was found to ameliorate the disease phenotype. Noting that there is a proven relationship between graft survival and amelioration of the disease course (reversal of motor deficits), the findings within the same study of only a small minority of graft TH+ve donor cell survival required further explanation. Indeed, >95% remained as neural precursors and hence the mechanism of functional improvement was likely not to be exogenous cellular replacement, but rather influences of the graft on endogenous cellular function (Redmond et al. 2007; Bjugstad et al. 2008). For reasons alluded to earlier, human pluripotent—including induced and embryonic—stem cells (hiPSCs/hESCs) have several attractive features in this context including self-renewal and capacity for predictable manipulation using extrinsic developmentally rationalized cues. However, when hESC-derived midbrain dopaminergic neurons are implanted into a primate model of PD, disappointingly no TH+ve cells were again found to survive at postmortem analysis (Wakeman et al. 2014). Against this background, the authenticity of midbrain dopaminergic specification was re-evaluated. Indeed, it was previously demonstrated through a series of elegant experiments that FOXA2 is a key transcription factor for the specification of authentic midbrain dopaminergic neurons (Kittappa et al. 2007). The Studer lab next conducted groundbreaking work that built on this discovery, where authentic midbrain dopaminergic neurons (generated by first specifying floorplate cells rather than progression through a neural rosette paradigm) were transplanted into rodent and primate models of Parkinson’s disease. Crucially, these interventions yielded both functional amelioration and survival of TH+ve/FOXA2+ve neurons 3 months post-transplant (Kriks et al. 2011). This represented a milestone achievement in this field. Subsequent work has considered implanting patient-specific hiPSC (rather than hESC)-derived midbrain dopaminergic (mDA) neurons. This was first tested in an MPTP primate model. Three months post-transplant, the mDA neurons mature in the host brain, retaining their “A9” molecular phenotype. It remains unresolved as to which source of dopaminergic neurons is optimal for transplantation into Parkinson’s disease patients, and the field stands to benefit from direct comparison of fetal, hESC-, and hiPSC-derived cellular grafts in the context of longitudinal clinical and pathological follow-up studies (Brundin et al. 2010).

Excitingly, the first hiPSC trial into human patients with age-related macular degeneration took place in 2014 where autologous hiPSC-derived retinal pigment epithelial cells were implanted (Chakradhar 2016). The intervention seemingly ameliorated progression in the first patient 1 year post-transplant, and no adverse effects were reported. However, quality checking of a second hiPSC line revealed mutations that were not seen in the somatic cells from which they were derived, consistent with previous reports (Gore et al. 2011). At this stage, the trial was suspended given unknown oncogenicity of the cell lines and costs of generating an autologous hiPSC line for each patient treated. The laboratory leading this work has confirmed that they will now turn their attention towards allogeneic hiPSCs (Garber 2015), which may indeed prove to be more cost-effective given that a total of approximately 150 preselected donors, (constituting a “haplobank”) could cater for the vast majority of the population in the UK (Taylor et al. 2012). The infrastructure here could follow basic design principles of blood banks, although an hiPSC haplobank would clearly require bespoke processes and quality control. It also presupposes equal potency between hiPSC lines, which is not always practically achieved (Hu et al. 2010; Boulting et al. 2011). Although the nervous system is an immunologically “privileged” site, the precise requirement for immunosuppression in neural grafting remains unresolved. There is therefore a balance to be struck between utilizing comprehensively characterized allogeneic lines versus “personalized” autologous hiPSCs; the former seems to be a more practically and financially viable option in the immediate future. Further caution is required when one considers the potential toxicity of hiPSC-based cellular therapy specifically in the context of tumor formation. Reprogramming of somatic cells often utilizes lentiviral or retroviral strategies, raising the issue of random integration within the human genome of the host and related oncological consequences (Howe et al. 2008; Okita et al. 2007). Indeed, the process of directed differentiation itself may trigger a resurgence of exogenous transgene transcription (Okita et al. 2007). Some of this risk can be mitigated by using non-integrating (and even non-viral) approaches to reprogramming. It is also possible that upon transplantation into a diseased environment, the hiPSC derivatives may be vulnerable to the disease for which they are being implanted. Such spread of disease into stem cell grafts has already been demonstrated in the context of fetal ventral mesencephalic transplantation in Parkinson’s disease (Li et al. 2008). Indeed, similar concerns may exist with autologous transplantation of hiPSC derivatives, especially in genetic diseases. Recent advances in genome editing can at least partially temper this concern, where a specific mutation can be “corrected” in patient-specific iPSCs prior to autologous transplantation. Several varieties of customized nucleases are now established for genome editing, including zinc finger nucleases (ZFNs), transcription activator effector nucleases (TALENs), and the CRISPR-Cas9 system (Urnov et al. 2010; Sander and Joung 2014; Joung and Sander 2013). Significant optimization in both the design and quality checking of these approaches provides some early promise for their practical utility in regenerative medicine (Tsai and Joung 2014).

Current evidence suggests that cellular therapies can restore structure and function to some degree, but in order to be clinically tractable, they must first be shown to be superior to the current standard of care. Given that no disease-modifying therapies currently exist for most neurodegenerative diseases, it is the hope that cellular strategies may slow down, halt, or ideally reverse disease progression. An interesting idea to consider here is combinatorial therapy (e.g., cellular transplant plus GDNF infusion in the case of Parkinson’s disease), which may be required to achieve clinically evident disease modification. Crucially, clinical trials need careful design including predefined end points, rigorous safety assessment, reproducible cellular derivation, tumorogenicity assays, and demonstration of preserved efficacy in the context of immunosuppression. To realize cellular therapies for neurodegenerative disease, it is clear that transparent international collaboration will be a key driving force.

## Concluding remarks

**Fig. 3:**
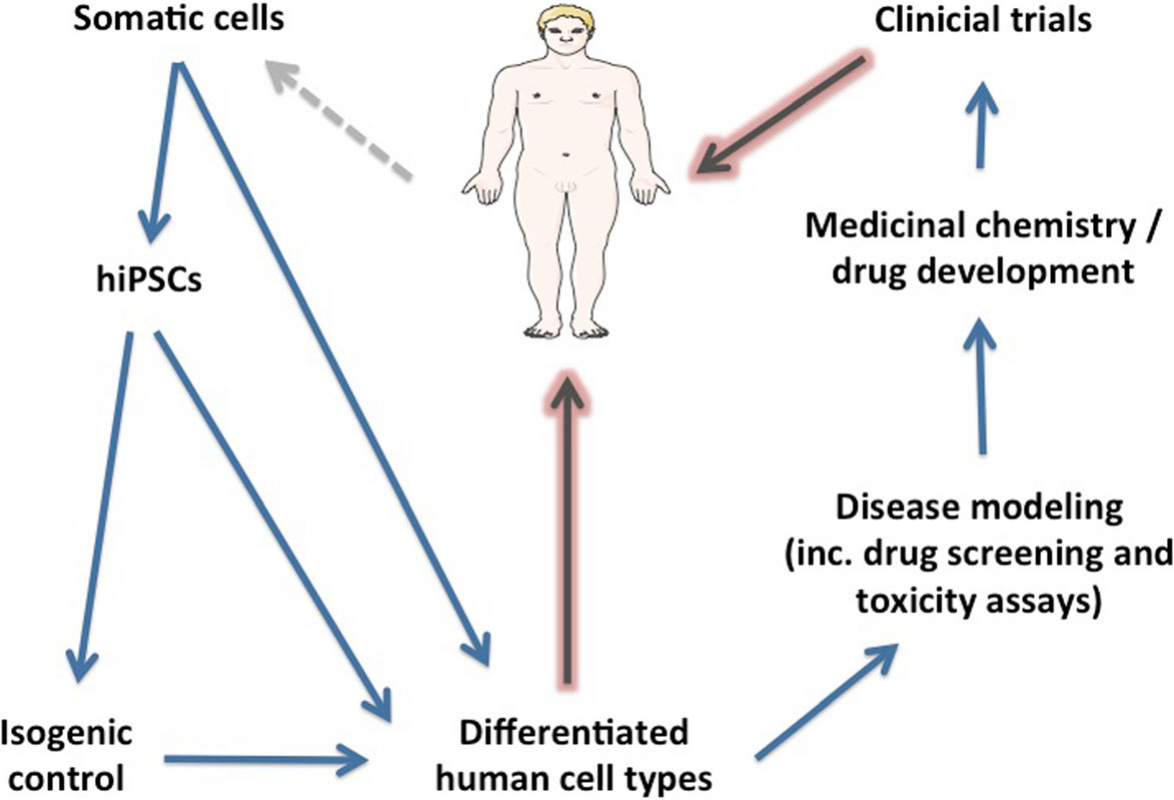
An organogram providing a framework within which human-induced pluripotent stem cells can be harnessed in the emerging discipline of regenerative neurology. Diagrams were drawn using templates freely available from Servier Medical Art (http://www.servier.co.uk/content/servier-medical-art)

HiPSCs hold tremendous promise for translational research in neurological disease (Connick et al. 2011). Although considerable advances have been made to date, these have not yet been optimally translated directly into improved patient care, which is the ultimate goal. Continuing advances in the directed differentiation of patient-specific hiPSCs into highly enriched populations of neuronal and glial subtypes will undoubtedly improve the precision of modeling cell type-specific phenotypes. There are certain regions of the neuraxis that have proved difficult to derive in vitro from hiPSCs such as the cerebellum (Wiethoff et al. 2015), although there have been recent promising advances (Wang et al. 2015). One important future goal is to conduct stage-defined transcriptome-wide analyses throughout human brain development from pluripotency through to adult old age and to include different neuraxial regions as they emerge and mature. This transcriptional atlas will, during its evolution, inform and guide directed differentiation strategies to multiple less well-studied neuraxial regions. Cell-based high-throughput screening methods for both ameliorating disease-specific phenotypes and toxicity assays will converge to reinforce a personalized approach to patient management. An important aspect to invest in for future studies is establishing salient (i.e., disease causing) phenotypes, which will necessitate discrimination of primary from secondary pathogenic events in time-resolved analyses. Functional genomic technologies with bespoke bioinformatics pipelines have proved powerful as unbiased approaches in elucidating transcriptional phenotypes, which can then be followed up in a hypothesis-driven manner. These advances coupled together with robust genome editing technologies are converging to help realize the promise of hiPSCs in regenerative neurology (see Fig. 3).

In future, we envisage diagnostic and therapeutic integration of patient-specific hiPSCs into clinical management. As a first step, this might involve rapid reprogramming and differentiation of patient-specific cells followed by a personalized regimen of drugs selected through high-throughput screening with concurrent toxicity assays. How soon this future can become a reality depends not only on scientific advances but also on practical and financial feasibility. The prospect of automating the majority of hiPSC culture is crucial to ensure scalability and reproducibility across experiments. This would also reduce cell culture demands on researchers and the risk of infection. Such technologies are now available, although wider usage is likely limited by cost. Clinically impactful advances in the hiPSC field will undoubtedly be realized sooner through cohesive international consortia and the closer collaboration/co-location of industry with both academic and clinical colleagues, in order to drive drug discovery and translational neuroscience towards an era of precision medicine.

## References

[CR1] Aflaki E, Borger DK, Moaven N, Stubblefield BK, Rogers SA, Patnaik S, Schoenen FJ, Westbroek W, Zheng W, Sullivan P, Fujiwara H, Sidhu R, Khaliq ZM, Lopez GJ, Goldstein DS, Ory DS, Marugan J, Sidransky E (2016). A new glucocerebrosidase chaperone reduces alpha-synuclein and glycolipid levels in iPSC-derived dopaminergic neurons from patients with Gaucher disease and parkinsonism. J Neurosci.

[CR2] Almeida S, Gascon E, Tran H, Chou HJ, Gendron TF, Degroot S, Tapper AR, Sellier C, Charlet-Berguerand N, Karydas A, Seeley WW, Boxer AL, Petrucelli L, Miller BL, Gao FB (2013). Modeling key pathological features of frontotemporal dementia with C9ORF72 repeat expansion in iPSC-derived human neurons. Acta Neuropathol.

[CR3] Athappily C, Patani R, Chawda S, Rosser E, De Silva R (2013). TS or not TS?. Pract Neurol.

[CR4] Bilican B, Serio A, Barmada SJ, Nishimura AL, Sullivan GJ, Carrasco M, Phatnani HP, Puddifoot CA, Story D, Fletcher J, Park IH, Friedman BA, Daley GQ, Wyllie DJ, Hardingham GE, Wilmut I, Finkbeiner S, Maniatis T, Shaw CE, Chandran S (2012). Mutant induced pluripotent stem cell lines recapitulate aspects of TDP-43 proteinopathies and reveal cell-specific vulnerability. Proc Natl Acad Sci U S A.

[CR5] Bjugstad KB, Teng YD, Redmond JR DE, Elsworth JD, Roth RH, Cornelius SK, Snyder EY, Sladek JR JR. Human neural stem cells migrate along the nigrostriatal pathway in a primate model of Parkinson’s disease. Exp Neurol. 2008;211:362–9. 10.1016/j.expneurol.2008.01.025 PMC2483423 18394605

[CR6] Boch J, Scholze H, Schornack S, Landgraf A, Hahn S, Kay S, Lahaye T, Nickstadt A, Bonas U (2009). Breaking the code of DNA binding specificity of TAL-type III effectors. Science.

[CR7] Booth LN, Brunet A (2016). The aging epigenome. Mol Cell.

[CR8] Boulting GL, Kiskinis E, Croft GF, Amoroso MW, Oakley DH, Wainger BJ, Williams DJ, Kahler DJ, Yamaki M, Davidow L, Rodolfa CT, Dimos JT, Mikkilineni S, Macdermott AB, Woolf CJ, Henderson CE, Wichterle H, Eggan K (2011). A functionally characterized test set of human induced pluripotent stem cells. Nat Biotechnol.

[CR9] Brundin P, Barker RA, Parmar M (2010). Neural grafting in Parkinson’s disease problems and possibilities. Prog Brain Res.

[CR10] Caiazzo M, Dell’anno MT, Dvoretskova E, Lazarevic D, Taverna S, Leo D, Sotnikova TD, Menegon A, Roncaglia P, Colciago G, Russo G, Carninci P, Pezzoli G, Gainetdinov RR, Gustincich S, Dityatev A, Broccoli V (2011). Direct generation of functional dopaminergic neurons from mouse and human fibroblasts. Nature.

[CR11] Caiazzo M, Giannelli S, Valente P, Lignani G, Carissimo A, Sessa A, Colasante G, Bartolomeo R, Massimino L, Ferroni S, Settembre C, Benfenati F, Broccoli V (2015). Direct conversion of fibroblasts into functional astrocytes by defined transcription factors. Stem Cell Reports.

[CR12] Chakradhar S (2016). An eye to the future: researchers debate best path for stem cell-derived therapies. Nat Med.

[CR13] Choi IY, Lim H, Estrellas K, Mula J, Cohen TV, Zhang Y, Donnelly CJ, Richard JP, Kim YJ, Kim H, Kazuki Y, Oshimura M, Li HL, Hotta A, Rothstein J, Maragakis N, Wagner KR, Lee G (2016). Concordant but varied phenotypes among Duchenne muscular dystrophy patient-specific myoblasts derived using a human iPSC-based model. Cell Rep.

[CR14] Clevers H (2016). Modeling development and disease with organoids. Cell.

[CR15] Cong L, Ran FA, Cox D, Lin S, Barretto R, Habib N, Hsu PD, Wu X, Jiang W, Marraffini LA, Zhang F (2013). Multiplex genome engineering using CRISPR/Cas systems. Science.

[CR16] Connick P, Patani R, Chandran S (2011). Stem cells as a resource for regenerative neurology. Pract Neurol.

[CR17] Consortium HDI (2012). Induced pluripotent stem cells from patients with Huntington’s disease show CAG-repeat-expansion-associated phenotypes. Cell Stem Cell.

[CR18] Devine MJ, Ryten M, Vodicka P, Thomson AJ, Burdon T, Houlden H, Cavaleri F, Nagano M, Drummond NJ, Taanman JW, Schapira AH, Gwinn K, Hardy J, Lewis PA, Kunath T (2011). Parkinson’s disease induced pluripotent stem cells with triplication of the alpha-synuclein locus. Nat Commun.

[CR19] Donnelly CJ, Zhang PW, Pham JT, Haeusler AR, Mistry NA, Vidensky S, Daley EL, Poth EM, Hoover B, Fines DM, Maragakis N, Tienari PJ, Petrucelli L, Traynor BJ, Wang J, Rigo F, Bennett CF, Blackshaw S, Sattler R, Rothstein JD (2013). RNA toxicity from the ALS/FTD C9ORF72 expansion is mitigated by antisense intervention. Neuron.

[CR20] Ebert AD, Yu J, Rose FF, Mattis VB, Lorson CL, Thomson JA, Svendsen CN (2009). Induced pluripotent stem cells from a spinal muscular atrophy patient. Nature.

[CR21] Egawa N, Kitaoka S, Tsukita K, Naitoh M, Takahashi K, Yamamoto T, Adachi F, Kondo T, Okita K, Asaka I, Aoi T, Watanabe A, Yamada Y, Morizane A, Takahashi J, Ayaki T, Ito H, Yoshikawa K, Yamawaki S, Suzuki S, Watanabe D, Hioki H, Kaneko T, Makioka K, Okamoto K, Takuma H, Tamaoka A, Hasegawa K, Nonaka T, Hasegawa M, Kawata A, Yoshida M, Nakahata T, Takahashi R, Marchetto MC, Gage FH, Yamanaka S, Inoue H (2012). Drug screening for ALS using patient-specific induced pluripotent stem cells. Sci Transl Med.

[CR22] Feldman EL, Boulis NM, Hur J, Johe K, Rutkove SB, Federici T, Polak M, Bordeau J, Sakowski SA, Glass JD (2014). Intraspinal neural stem cell transplantation in amyotrophic lateral sclerosis: phase 1 trial outcomes. Ann Neurol.

[CR23] Garber K (2015). RIKEN suspends first clinical trial involving induced pluripotent stem cells. Nat Biotechnol.

[CR24] Glass JD, Boulis NM, Johe K, Rutkove SB, Federici T, Polak M, Kelly C, Feldman EL (2012). Lumbar intraspinal injection of neural stem cells in patients with amyotrophic lateral sclerosis: results of a phase I trial in 12 patients. Stem Cells.

[CR25] Goldman SA, Kuypers NJ (2015). How to make an oligodendrocyte. Development.

[CR26] Gonzalez F, Boue S, Izpisua Belmonte JC (2011). Methods for making induced pluripotent stem cells: reprogramming a la carte. Nat Rev Genet.

[CR27] Gore A, Li Z, Fung HL, Young JE, Agarwal S, Antosiewicz-Bourget J, Canto I, Giorgetti A, Israel MA, Kiskinis E, Lee JH, Loh YH, Manos PD, Montserrat N, Panopoulos AD, Ruiz S, Wilbert ML, Yu J, Kirkness EF, Izpisua Belmonte JC, Rossi DJ, Thomson JA, Eggan K, Daley GQ, Goldstein LS, Zhang K (2011). Somatic coding mutations in human induced pluripotent stem cells. Nature.

[CR28] Greenhough S, Medine CN, Hay DC (2010). Pluripotent stem cell derived hepatocyte like cells and their potential in toxicity screening. Toxicology.

[CR29] Grunseich C, Zukosky K, Kats IR, Ghosh L, Harmison GG, Bott LC, Rinaldi C, Chen KL, Chen G, Boehm M, Fischbeck KH (2014). Stem cell-derived motor neurons from spinal and bulbar muscular atrophy patients. Neurobiol Dis.

[CR30] Hallett PJ, Deleidi M, Astradsson A, Smith GA, Cooper O, Osborn TM, Sundberg M, Moore MA, Perez-TORRES E, Brownell AL, Schumacher JM, Spealman RD, Isacson O (2015). Successful function of autologous iPSC-derived dopamine neurons following transplantation in a non-human primate model of Parkinson’s disease. Cell Stem Cell.

[CR31] Han Y, Miller A, Mangada J, Liu Y, Swistowski A, Zhan M, Rao MS, Zeng X (2009). Identification by automated screening of a small molecule that selectively eliminates neural stem cells derived from hESCs but not dopamine neurons. PLoS One.

[CR32] Hendriks WT, Warren CR, Cowan CA (2016). Genome editing in human pluripotent stem cells: approaches, pitfalls, and solutions. Cell Stem Cell.

[CR33] Ho, R., Sances, S., Gowing, G., Amoroso, M. W., O’rourke, J. G., Sahabian, A., Wichterle, H., Baloh, R. H., Sareen, D. & Svendsen, C. N.. ALS disrupts spinal motor neuron maturation and aging pathways within gene co-expression networks. *Nat Neurosci*. 2016 10.1038/nn.4345 PMC5003654 27428653

[CR34] Howe SJ, Mansour MR, Schwarzwaelder K, Bartholomae C, Hubank M, Kempski H, Brugman MH, Pike-Overzet K, Chatters SJ, De Ridder D, Gilmour KC, Adams S, Thornhill SI, Parsley KL, Staal FJ, Gale RE, Linch DC, Bayford J, Brown L, Quaye M, Kinnon C, Ancliff P, Webb DK, Schmidt M, Von Kalle C, Gaspar HB, Thrasher AJ (2008). Insertional mutagenesis combined with acquired somatic mutations causes leukemogenesis following gene therapy of SCID-X1 patients. J Clin Invest.

[CR35] Hu BY, Weick JP, Yu J, Ma LX, Zhang XQ, Thomson JA, Zhang SC (2010). Neural differentiation of human induced pluripotent stem cells follows developmental principles but with variable potency. Proc Natl Acad Sci U S A.

[CR36] Joung JK, Sander JD (2013). TALENs: a widely applicable technology for targeted genome editing. Nat Rev Mol Cell Biol.

[CR37] Kaufmann M, Schuffenhauer A, Fruh I, Klein J, Thiemeyer A, Rigo P, Gomez-Mancilla B, Heidinger-Millot V, Bouwmeester T, Schopfer U, Mueller M, Fodor BD, Cobos-Correa A (2015). High-throughput screening using iPSC-derived neuronal progenitors to identify compounds counteracting epigenetic gene silencing in fragile X syndrome. J Biomol Screen.

[CR38] Kiskinis E, Sandoe J, Williams LA, Boulting GL, Moccia R, Wainger BJ, Han S, Peng T, Thams S, Mikkilineni S, Mellin C, Merkle FT, Davis-Dusenbery BN, Ziller M, Oakley D, Ichida J, Di Costanzo S, Atwater N, Maeder ML, Goodwin MJ, Nemesh J, Handsaker RE, Paull D, Noggle S, Mccarroll SA, Joung JK, Woolf CJ, Brown RH, Eggan K (2014). Pathways disrupted in human ALS motor neurons identified through genetic correction of mutant SOD1. Cell Stem Cell.

[CR39] Kittappa R, Chang WW, Awatramani RB, Mckay RD (2007). The foxa2 gene controls the birth and spontaneous degeneration of dopamine neurons in old age. PLoS Biol.

[CR40] Kondo T, Asai M, Tsukita K, Kutoku Y, Ohsawa Y, Sunada Y, Imamura K, Egawa N, Yahata N, Okita K, Takahashi K, Asaka I, Aoi T, Watanabe A, Watanabe K, Kadoya C, Nakano R, Watanabe D, Maruyama K, Hori O, Hibino S, Choshi T, Nakahata T, Hioki H, Kaneko T, Naitoh M, Yoshikawa K, Yamawaki S, Suzuki S, Hata R, Ueno S, Seki T, Kobayashi K, Toda T, Murakami K, Irie K, Klein WL, Mori H, Asada T, Takahashi R, Iwata N, Yamanaka S, Inoue H (2013). Modeling Alzheimer’s disease with iPSCs reveals stress phenotypes associated with intracellular Abeta and differential drug responsiveness. Cell Stem Cell.

[CR41] Kriks S, Shim JW, Piao J, Ganat YM, Wakeman DR, Xie Z, Carrillo-Reid L, Auyeung G, Antonacci C, Buch A, Yang L, Beal MF, Surmeier DJ, Kordower JH, Tabar V, Studer L (2011). Dopamine neurons derived from human ES cells efficiently engraft in animal models of Parkinson’s disease. Nature.

[CR42] Kumari D, Swaroop M, Southall N, Huang W, Zheng W, Usdin K (2015). High-throughput screening to identify compounds that increase fragile X mental retardation protein expression in neural stem cells differentiated from fragile X syndrome patient-derived induced pluripotent stem cells. Stem Cells Transl Med.

[CR43] Lebkowski J (2011). GRNOPC1: the world’s first embryonic stem cell-derived therapy. Interview with Jane Lebkowski. Regen Med.

[CR44] Lee G, Papapetrou EP, Kim H, Chambers SM, Tomishima MJ, Fasano CA, Ganat YM, Menon J, Shimizu F, Viale A, Tabar V, Sadelain M, Studer L (2009). Modelling pathogenesis and treatment of familial dysautonomia using patient-specific iPSCs. Nature.

[CR45] Lee G, Ramirez CN, Kim H, Zeltner N, Liu B, Radu C, Bhinder B, Kim YJ, Choi IY, Mukherjee-Clavin B, Djaballah H, Studer L (2012). Large-scale screening using familial dysautonomia induced pluripotent stem cells identifies compounds that rescue IKBKAP expression. Nat Biotechnol.

[CR46] Li JY, Englund E, Holton JL, Soulet D, Hagell P, Lees AJ, Lashley T, Quinn NP, Rehncrona S, Bjorklund A, Widner H, Revesz T, Lindvall O, Brundin P (2008). Lewy bodies in grafted neurons in subjects with Parkinson’s disease suggest host-to-graft disease propagation. Nat Med.

[CR47] Liu GH, Qu J, Suzuki K, Nivet E, Li M, Montserrat N, Yi F, Xu X, Ruiz S, Zhang W, Wagner U, Kim A, Ren B, Li Y, Goebl A, Kim J, Soligalla RD, Dubova I, Thompson J, Yates J, Esteban CR, Sancho-Martinez I, Izpisua Belmonte JC (2012). Progressive degeneration of human neural stem cells caused by pathogenic LRRK2. Nature.

[CR48] Liu Y, Lopez-Santiago LF, Yuan Y, Jones JM, Zhang H, O’malley HA, Patino GA, O’brien JE, Rusconi R, Gupta A, Thompson RC, Natowicz MR, Meisler MH, Isom LL, Parent JM (2013). Dravet syndrome patient-derived neurons suggest a novel epilepsy mechanism. Ann Neurol.

[CR49] Malik N, Efthymiou AG, Mather K, Chester N, Wang X, Nath A, Rao MS, Steiner JP (2014). Compounds with species and cell type specific toxicity identified in a 2000 compound drug screen of neural stem cells and rat mixed cortical neurons. Neurotoxicology.

[CR50] Marchetto MC, Carromeu C, Acab A, Yu D, Yeo GW, Mu Y, Chen G, Gage FH, Muotri AR (2010). A model for neural development and treatment of Rett syndrome using human induced pluripotent stem cells. Cell.

[CR51] Maury Y, Come J, Piskorowski RA, Salah-Mohellibi N, Chevaleyre V, Peschanski M, Martinat C, Nedelec S (2015). Combinatorial analysis of developmental cues efficiently converts human pluripotent stem cells into multiple neuronal subtypes. Nat Biotechnol.

[CR52] Medda X, Mertens L, Versweyveld S, Diels A, Barnham L, Bretteville A, Buist A, Verheyen A, Royaux I, Ebneth A, Cabrera-Socorro A (2016). Development of a scalable, high-throughput-compatible assay to detect tau aggregates using iPSC-derived cortical neurons maintained in a three-dimensional culture format. J Biomol Screen.

[CR53] Merkle FT, Eggan K (2013). Modeling human disease with pluripotent stem cells: from genome association to function. Cell Stem Cell.

[CR54] Mertens J, Paquola AC, Ku M, Hatch E, Bohnke L, Ladjevardi S, Mcgrath S, Campbell B, Lee H, Herdy JR, Goncalves JT, Toda T, Kim Y, Winkler J, Yao J, Hetzer MW, Gage FH (2015). Directly reprogrammed human neurons retain aging-associated Transcriptomic signatures and reveal age-related nucleocytoplasmic defects. Cell Stem Cell.

[CR55] Miller JD, Ganat YM, Kishinevsky S, Bowman RL, Liu B, Tu EY, Mandal PK, Vera E, Shim JW, Kriks S, Taldone T, Fusaki N, Tomishima MJ, Krainc D, Milner TA, Rossi DJ, Studer L (2013). Human iPSC-based modeling of late-onset disease via progerin-induced aging. Cell Stem Cell.

[CR56] Mishra, H. K., Prots, I., Havlicek, S., Kohl, Z., Perez-Branguli, F., Boerstler, T., Anneser, L., Minakaki, G., Wend, H., Hampl, M., Leone, M., Bruckner, M., Klucken, J., Reis, A., Boyer, L., Schuierer, G., Behrens, J., Lampert, A., Engel, F. B., Gage, F. H., Winkler, J. & Winner, B. . GSK3ss-dependent dysregulation of neurodevelopment in SPG11-patient iPSC model. Ann Neurol. 2016 10.1002/ana.24633 PMC5084783 26971897

[CR57] Mu S, Wang J, Zhou G, Peng W, He Z, Zhao Z, Mo C, Qu J, Zhang J (2014). Transplantation of induced pluripotent stem cells improves functional recovery in Huntington’s disease rat model. PLoS One.

[CR58] Neofytou E, O’brien CG, Couture LA, Wu JC (2015). Hurdles to clinical translation of human induced pluripotent stem cells. J Clin Invest.

[CR59] Ng SY, Soh BS, Rodriguez-Muela N, Hendrickson DG, Price F, Rinn JL, Rubin LL (2015). Genome-wide RNA-Seq of human motor neurons implicates selective ER stress activation in spinal muscular atrophy. Cell Stem Cell.

[CR60] Nguyen HN, Byers B, Cord B, Shcheglovitov A, Byrne J, Gujar P, Kee K, Schule B, Dolmetsch RE, Langston W, Palmer TD, Pera RR (2011). LRRK2 mutant iPSC-derived DA neurons demonstrate increased susceptibility to oxidative stress. Cell Stem Cell.

[CR61] Nizzardo, M., Bucchia, M., Ramirez, A., Trombetta, E., Bresolin, N., Comi, G. P. & Corti, S.. iPSC-derived LewisX+CXCR4+beta1-integrin+ neural stem cells improve the amyotrophic lateral sclerosis phenotype by preserving motor neurons and muscle innervation in human and rodent models. Hum Mol Genet*.* 2016 10.1093/hmg/ddw163 27270413

[CR62] Nizzardo M, Simone C, Rizzo F, Ruggieri M, Salani S, Riboldi G, Faravelli I, Zanetta C, Bresolin N, Comi GP, Corti S (2014). Minimally invasive transplantation of iPSC-derived ALDHhiSSCloVLA4+ neural stem cells effectively improves the phenotype of an amyotrophic lateral sclerosis model. Hum Mol Genet.

[CR63] Oh Y, Cho GS, Li Z, Hong I, Zhu R, Kim MJ, Kim YJ, Tampakakis E, Tung L, Huganir R, Dong X, Kwon C, Lee G (2016). Functional coupling with cardiac muscle promotes maturation of hPSC-derived sympathetic neurons. Cell Stem Cell.

[CR64] Okita K, Ichisaka T, Yamanaka S (2007). Generation of germline-competent induced pluripotent stem cells. Nature.

[CR65] Patani R (2016). Generating diverse spinal motor neuron subtypes from human pluripotent stem cells. Stem Cells Int.

[CR66] Patani R, Lewis PA, Trabzuni D, Puddifoot CA, Wyllie DJ, Walker R, Smith C, Hardingham GE, Weale M, Hardy J, Chandran S, Ryten M (2012). Investigating the utility of human embryonic stem cell-derived neurons to model ageing and neurodegenerative disease using whole-genome gene expression and splicing analysis. J Neurochem.

[CR67] Patani R, Muhammed N, Chaudhuri A (2013). Flexor hallucis brevis spasm. Muscle Nerve.

[CR68] Patani R, Sibley CR, Chandran S, Ule J (2012). Using human pluripotent stem cells to study post-transcriptional mechanisms of neurodegenerative diseases. Brain Res.

[CR69] Peng J, Liu Q, Rao MS, Zeng X (2013). Using human pluripotent stem cell-derived dopaminergic neurons to evaluate candidate Parkinson’s disease therapeutic agents in MPP+ and rotenone models. J Biomol Screen.

[CR70] Redmond Jr DE, Bjugstad KB, Teng YD, Ourednik V, Ourednik J, Wakeman DR, Parsons XH, Gonzalez R, Blanchard BC, Kim SU, Gu Z, Lipton SA, Markakis EA, Roth RH, Elsworth JD, Sladek JR JR, Sidman RL, Snyder EY. Behavioral improvement in a primate Parkinson’s model is associated with multiple homeostatic effects of human neural stem cells. Proc Natl Acad Sci U S A. 2007;104:12175–80. 10.1073/pnas.0704091104 PMC1896134 17586681

[CR71] Reinhardt P, Schmid B, Burbulla LF, Schondorf DC, Wagner L, Glatza M, Hoing S, Hargus G, Heck SA, Dhingra A, Wu G, Muller S, Brockmann K, Kluba T, Maisel M, Kruger R, Berg D, Tsytsyura Y, Thiel CS, Psathaki OE, Klingauf J, Kuhlmann T, Klewin M, Muller H, Gasser T, Scholer HR, Sterneckert J (2013). Genetic correction of a LRRK2 mutation in human iPSCs links parkinsonian neurodegeneration to ERK-dependent changes in gene expression. Cell Stem Cell.

[CR72] Ren Y, Jiang H, Hu Z, Fan K, Wang J, Janoschka S, Wang X, Ge S, Feng J (2015). Parkin mutations reduce the complexity of neuronal processes in iPSC-derived human neurons. Stem Cells.

[CR73] Rigamonti A, Repetti GG, Sun C, Price FD, Reny DC, Rapino F, Weisinger K, Benkler C, Peterson QP, Davidow LS, Hansson EM, Rubin LL (2016). Large-scale production of mature neurons from human pluripotent stem cells in a three-dimensional suspension culture system. Stem Cell Reports.

[CR74] Ringel M, Tollman P, Hersch G, Schulze U (2013). Does size matter in R&D productivity? If not, what does?. Nat Rev Drug Discov.

[CR75] Rubin LL (2008). Stem cells and drug discovery: the beginning of a new era?. Cell.

[CR76] Ryan KR, Sirenko O, Parham F, Hsieh JH, Cromwell EF, Tice RR, Behl M (2016). Neurite outgrowth in human induced pluripotent stem cell-derived neurons as a high-throughput screen for developmental neurotoxicity or neurotoxicity. Neurotoxicology.

[CR77] Samani A, Davagnanam I, Cockerell OC, Ramsay A, Patani R, Chataway J (2015). Lymphomatosis cerebri: a treatable cause of rapidly progressive dementia. J Neurol Neurosurg Psychiatry.

[CR78] Sander JD, Joung JK (2014). CRISPR-Cas systems for editing, regulating and targeting genomes. Nat Biotechnol.

[CR79] Sanders LH, Laganiere J, Cooper O, Mak SK, Vu BJ, Huang YA, Paschon DE, Vangipuram M, Sundararajan R, Urnov FD, Langston JW, Gregory PD, Zhang HS, Greenamyre JT, Isacson O, Schule B (2014). LRRK2 mutations cause mitochondrial DNA damage in iPSC-derived neural cells from Parkinson’s disease patients: reversal by gene correction. Neurobiol Dis.

[CR80] Scannell JW, Blanckley A, Boldon H, Warrington B (2012). Diagnosing the decline in pharmaceutical R&D efficiency. Nat Rev Drug Discov.

[CR81] Schondorf DC, Aureli M, Mcallister FE, Hindley CJ, Mayer F, Schmid B, Sardi SP, Valsecchi M, Hoffmann S, Schwarz LK, Hedrich U, Berg D, Shihabuddin LS, Hu J, Pruszak J, Gygi SP, Sonnino S, Gasser T, Deleidi M (2014). iPSC-derived neurons from GBA1-associated Parkinson’s disease patients show autophagic defects and impaired calcium homeostasis. Nat Commun.

[CR82] Scott CT, Magnus D (2014). Wrongful termination: lessons from the Geron clinical trial. Stem Cells Transl Med.

[CR83] Seok J, Warren HS, Cuenca AG, Mindrinos MN, Baker HV, Xu W, Richards DR, Mcdonald-Smith GP, Gao H, Hennessy L, Finnerty CC, Lopez CM, Honari S, Moore EE, Minei JP, Cuschieri J, Bankey PE, Johnson JL, Sperry J, Nathens AB, Billiar TR, West MA, Jeschke MG, Klein MB, Gamelli RL, Gibran NS, Brownstein BH, Miller-Graziano C, Calvano SE, Mason PH, Cobb JP, Rahme LG, Lowry SF, Maier RV, Moldawer LL, Herndon DN, Davis RW, Xiao W, Tompkins RG, INFLAMMATION & HOST RESPONSE TO INJURY, L. S. C. R. P (2013). Genomic responses in mouse models poorly mimic human inflammatory diseases. Proc Natl Acad Sci U S A.

[CR84] Serio A, Bilican B, Barmada SJ, Ando DM, Zhao C, Siller R, Burr K, Haghi G, Story D, Nishimura AL, Carrasco MA, Phatnani HP, Shum C, Wilmut I, Maniatis T, Shaw CE, Finkbeiner S, Chandran S (2013). Astrocyte pathology and the absence of non-cell autonomy in an induced pluripotent stem cell model of TDP-43 proteinopathy. Proc Natl Acad Sci U S A.

[CR85] Shribman, S., Patani, R., Deeb, J. & Chaudhuri, A.. Voltage-gated potassium channelopathy: an expanding spectrum of clinical phenotypes. BMJ Case Rep*,*2013 2013. 10.1136/bcr-2012-007742 PMC3603777 23314449

[CR86] Siller R, Greenhough S, Naumovska E, Sullivan GJ (2015). Small-molecule-driven hepatocyte differentiation of human pluripotent stem cells. Stem Cell Rep.

[CR87] Son EY, Ichida JK, Wainger BJ, Toma JS, Rafuse VF, Woolf CJ, Eggan K (2011). Conversion of mouse and human fibroblasts into functional spinal motor neurons. Cell Stem Cell.

[CR88] Takahashi K, Tanabe K, Ohnuki M, Narita M, Ichisaka T, Tomoda K, Yamanaka S (2007). Induction of pluripotent stem cells from adult human fibroblasts by defined factors. Cell.

[CR89] Takahashi K, Yamanaka S (2006). Induction of pluripotent stem cells from mouse embryonic and adult fibroblast cultures by defined factors. Cell.

[CR90] Taylor CJ, Peacock S, Chaudhry AN, Bradley JA, Bolton EM (2012). Generating an iPSC bank for HLA-matched tissue transplantation based on known donor and recipient HLA types. Cell Stem Cell.

[CR91] Thiruvalluvan, A., Czepiel, M., Kap, Y. A., Mantingh-OTTER, I., Vainchtein, I., Kuipers, J., Bijlard, M., Baron, W., Giepmans, B., Bruck, W., Thart, B. A., Boddeke, E. & Copray, S. 2016. Survival and functionality of human induced pluripotent stem cell-derived oligodendrocytes in a nonhuman primate model for multiple sclerosis. Stem *Cells Transl Med.* 10.5966/sctm.2016-0024 PMC5070510 27400790

[CR92] Tsai SQ, Joung JK (2014). What’s changed with genome editing?. Cell Stem Cell.

[CR93] Tyzack G, Lakatos A, Patani R (2016). Human stem cell-derived astrocytes: specification and relevance for neurological disorders. Curr Stem Cell Rep.

[CR94] Urnov FD, Rebar EJ, Holmes MC, Zhang HS, Gregory PD (2010). Genome editing with engineered zinc finger nucleases. Nat Rev Genet.

[CR95] Wainger BJ, Kiskinis E, Mellin C, Wiskow O, Han SS, Sandoe J, Perez NP, Williams LA, Lee S, Boulting G, Berry JD, Brown RH, Cudkowicz ME, Bean BP, Eggan K, Woolf CJ (2014). Intrinsic membrane hyperexcitability of amyotrophic lateral sclerosis patient-derived motor neurons. Cell Rep.

[CR96] Wakeman DR, Weiss S, Sladek JR, Elsworth JD, Bauereis B, Leranth C, Hurley PJ, Roth RH, Redmond DE (2014). Survival and integration of neurons derived from human embryonic stem cells in MPTP-lesioned primates. Cell Transplant.

[CR97] Wang S, Wang B, Pan N, Fu L, Wang C, Song G, An J, Liu Z, Zhu W, Guan Y, Xu ZQ, Chan P, Chen Z, Zhang YA (2015). Differentiation of human induced pluripotent stem cells to mature functional Purkinje neurons. Sci Rep.

[CR98] Wiethoff S, Arber C, Li A, Wray S, Houlden H, Patani R (2015). Using human induced pluripotent stem cells to model cerebellar disease: hope and hype. J Neurogenet.

[CR99] Wood AJ, Lo TW, Zeitler B, Pickle CS, Ralston EJ, Lee AH, Amora R, Miller JC, Leung E, Meng X, Zhang L, Rebar EJ, Gregory PD, Urnov FD, Meyer BJ (2011). Targeted genome editing across species using ZFNs and TALENs. Science.

[CR100] Xie N, Tang B (2016). The application of human iPSCs in neurological diseases: from bench to bedside. Stem Cells Int.

[CR101] Xu M, Lee EM, Wen Z, Cheng Y, Huang WK, Qian X, Tcw J, Kouznetsova J, Ogden SC, Hammack C, Jacob F, Nguyen HN, Itkin M, Hanna C, Shinn P, Allen C, Michael SG, Simeonov A, Huang W, Christian KM, Goate A, Brennand KJ, Huang R, Xia M, Ming GL, Zheng W, Song H, Tang H (2016). Identification of small-molecule inhibitors of Zika virus infection and induced neural cell death via a drug repurposing screen. Nat Med.

[CR102] Yagi T, Ito D, Okada Y, Akamatsu W, Nihei Y, Yoshizaki T, Yamanaka S, Okano H, Suzuki N (2011). Modeling familial Alzheimer’s disease with induced pluripotent stem cells. Hum Mol Genet.

[CR103] Yang N, Zuchero JB, Ahlenius H, Marro S, Ng YH, Vierbuchen T, Hawkins JS, Geissler R, Barres BA, Wernig M (2013). Generation of oligodendroglial cells by direct lineage conversion. Nat Biotechnol.

[CR104] Yang YM, Gupta SK, Kim KJ, Powers BE, Cerqueira A, Wainger BJ, Ngo HD, Rosowski KA, Schein PA, Ackeifi CA, Arvanites AC, Davidow LS, Woolf CJ, Rubin LL (2013). A small molecule screen in stem-cell-derived motor neurons identifies a kinase inhibitor as a candidate therapeutic for ALS. Cell Stem Cell.

[CR105] Yoon KJ, Nguyen HN, Ursini G, Zhang F, Kim NS, Wen Z, Makri G, Nauen D, Shin JH, Park Y, Chung R, Pekle E, Zhang C, Towe M, Hussaini SM, Lee Y, Rujescu D, St Clair D, Kleinman JE, Hyde TM, Krauss G, Christian KM, Rapoport JL, Weinberger DR, Song H, Ming GL (2014). Modeling a genetic risk for schizophrenia in iPSCs and mice reveals neural stem cell deficits associated with adherens junctions and polarity. Cell Stem Cell.

[CR106] Zhang, C., Cao, J., Li, X., Xu, H., Wang, W., Wang, L., Zhao, X., Li, W., Jiao, J., Hu, B., Zhou, Q. & Zhao, T.. Treatment of multiple sclerosis by transplantation of neural stem cells derived from induced pluripotent stem cells. Sci China Life Sci. 2016 10.1007/s11427-016-0114-9 27233903

[CR107] Zhang N, An MC, Montoro D, Ellerby LM (2010). Characterization of human Huntington’s disease cell model from induced pluripotent stem cells. PLoS Curr.

[CR108] Zhao HW, Gu XQ, Chailangkarn T, Perkins G, Callacondo D, Appenzeller O, Poulsen O, Zhou D, muotri AR, Haddad GG (2015). Altered iPSC-derived neurons’ sodium channel properties in subjects with Monge’s disease. Neuroscience.

[CR109] Zirra A, Wiethoff S, Patani R (2016). Neural conversion and patterning of human pluripotent stem cells: a developmental perspective. Stem Cells Int.

